# Influence of Minor Zn Addition on Precipitation Behavior and Intergranular Corrosion Properties of Al-Mg-Si Alloy

**DOI:** 10.3390/ma13030650

**Published:** 2020-02-01

**Authors:** Shuiqing Chi, Yunlai Deng, Xuehong Xu, Xiaobin Guo

**Affiliations:** 1 School of Materials Science and Engineering, Central South University, Changsha 410083, China; chishuiqing@csu.edu.cn (S.C.); luckdeng@csu.edu.cn (Y.D.); 2 Light Alloy Research Institute, Central South University, Changsha 410083, China; 173801014@csu.edu.cn

**Keywords:** Al-Mg-Si alloy, zinc, microstructure, intergranular corrosion, mechanical property

## Abstract

The effect of 0.2 wt.% Zn addition on microstructure, age hardening and intergranular corrosion (IGC) properties of Al-Mg-Si alloy were investigated by scanning electron microscope, transmission electron microscope, hardness testing, and electrochemistry testing. The results showed that the addition of Zn can accelerate the transformation of GP zones into β″, and make the intragranular precipitates become smaller and with higher density. This is beneficial to the precipitation strengthening of the alloy, leading to obtaining higher hardness and enhancing the age hardening response. The peak hardness of the alloy with the addition of Zn is 125.8 HV which means increasing the hardness by 12.7 HV, compared with the alloy without Zn. However, the addition of Zn makes the precipitate-free zone (PFZ) of the alloy wider, and coarsens the grain boundary precipitates slightly, which result in the reduction of IGC resistance of Al-Mg-Si alloy. The maximum corrosion depth of the Zn-containing alloy is 121.3 μm in the peak age condition, which is 35.7 μm deeper than the alloy without Zn. The result of the potentiodynamic polarization curves also demonstrated the increase of IGC sensitivity. The corrosion current density of the alloy with added Zn is 0.595 μA/cm^2^ in the peak age condition, while that for the alloy without Zn is 0.199 μA/cm^2^.

## 1. Introduction

As heat-treatable alloys, Al-Mg-Si alloys have been widely used in automobile manufacturing, and have become the preferred material for automobile body plate, due to their high specific strength, good formability, excellent corrosion resistance, and weldability [1,2]. However, these alloys are still face some challenges regarding their application, such as how to further improve their corrosion resistance, moldability, and get a quick age hardening response after a short-time paint baking [3]. Intergranular corrosion (IGC) can be observed in Al-Mg-Si alloys, which is influenced by the composition and the different heat-treatment conditions. Some studies show that the addition of Cu in the Al-Mg-Si alloys can enhance the age hardening response, but at the same time increasing the corrosion susceptibility. Moreover, the higher Cu content, the more sensitive to intergranular corrosion [4]. The addition of excessive Si in the alloy will lead to better mechanical properties. However, the precipitate-free zone (PFZ) will dissolve as the anode, due to the Si particles precipitated in the grain boundary. Al-Mg-Si alloys with excessive Mg can have good corrosion properties, but their age hardening response will be lower [5]. The IGC of 6061 aluminum alloys showed susceptibility at under aged state, and pitting was the dominant corrosion mode for the over aged state, and the alloys showed maximum susceptibility to IGC at peak aged state [6]. However, it was shown in [7] that the Al-Mg-Si-Cu alloys become susceptible to IGC again when the samples were heavily over aged. It was dependent on the precipitation of the closely-neighbored grain boundary Q-phase precipitates which accelerated the dissolution of the PFZ as anode. Additionally, Wang et al. [8] founded that after two-step aging treatment (180 °C/2 h + 160 °C/120 h), Al-Mg-Si-Cu alloys can get microstructure with a high number density β″ precipitates and discretely distributed grain boundary precipitates, so that the alloys can show a higher IGC resistance without strength loss.

Compared to Al-Mg-Si alloys, Al-Zn-Mg alloys have been widely used in aerospace, because of their high strength. The precipitation sequence of Al-Zn-Mg alloys is: super-saturated solid solution (SSSS) → Guinier–Preston (GP) zones → η′ → η (MgZn_2_) [9]. The η’ phases have been considered as the main strengthening precipitates of Al-Zn-Mg alloys at the peak age state. Furthermore, the atomic radius of Zn atom is close to that of Al atom, which leads to the low lattice distortion. Therefore, under the premise of ensuring the good forming ability of the alloy, the age hardening response of Al-Mg-Si alloy can be improved by adding Zn atoms. Recently, studies on Zn addition in Al-Mg-Si alloys have been reported by many researchers: Saito et al. [10] found that addition of Zn did not change the precipitation sequence of Al-Mg-Si alloys, which still was SSSS → Si clusters and Mg clusters → dissolution of Mg clusters → Mg, Si co-clusters → GP zones → β″ → β′ → β [11]. Cai et al. [12] observed that addition of Zn helps produce faster and higher age hardening and reduce the electrical conductivity at the beginning of aging. Ding et al. [13] suggested that the precipitation of η′ phases and GP (II) zones of η-MgZn_2_ improves the age hardening response of the Al-Mg-Si-Zn alloy. Guo et al. [14] pointed out that Zn added in Al-0.6Mg-0.9Si-0.2Cu alloys can be used as nucleation sites of Mg-Si phases, which is conducive to the formation of small and dense Mg-Si phases and accelerates age hardening. To date, most of the works in the literature focus on the effects of Zn addition more than 1.0 wt.% on the microstructure and mechanical properties, there are short of the studies for the influence of minor Zn addition on the microstructure, mechanical properties and IGC properties. This study aims to analyze the effects of minor Zn additions on the microstructure and properties of Al-Mg-Si alloys. This is of great significance for composition optimization design, material preparation and application of Al-Mg-Si alloys.

## 2. Experimental

The material used in this experiment is aluminum alloy rolled sheet with a thickness of 3 mm. Two different alloys where studied, AA and AZ (Al-Mg-Si alloys with and without Zn addition, respectively), with chemical composition as shown in Table 1. The solution treatment was conducted at 550 °C for 60 min, and then water quenched to room temperature [15]. Subsequently, the samples were artificially aged at 150, 170, 190, and 210 °C for 24 h, respectively. 

In order to measure the age hardening curves of those samples, tests with 200HVS-5 Vickers hardness tester (Laizhou Lyric Testing Equipment Co., Ltd., Laizhou, China) were carried out at a load of 3 kg with a dwell time of 15 s. Each sample was test five times, and the average hardness values were provided. The tensile properties of the alloys were measured by DDL-100 tensile testing machine (Changchun Institute of Mechanical Sciences, Changchun, China) at room temperature, the strain rate approximately was 5 × 10^−4^ s^−1^. The tensile specimens were prepared according to GB/T 228.1-2010 [16]. The samples were machined from the rolled sheets and the direction was perpendicular to the rolling direction, Tensile fractures of alloys were observed on ZEISS M10A scanning electron microscope (SEM, Oberkochen, Germany) equipped with X-ray energy dispersive spectrometers (EDS) system, operated at 20 kV. 

The IGC behavior was investigated by performing continuous immersion tests (IGC test) according to the GB/T 7998—2005 [17] method. The samples at different aged conditions were etched at 10% sodium hydroxide solution for 15 min, and then cleaned at 30% nitric acid, followed by immersion into the solution of 10 mL/L (30%) hydrogen peroxide and 57 g/L sodium chloride.at 35 °C for 24 h. The corrosion morphology of the cross section was observed by ZEISS M10A scanning electron microscope. The electrochemical tests were carried out by Multi Autolab M204 (Metrohm Autolab, Utrecht, the Netherlands) with a three-electrode cell: the reference electrode was saturated calomel electrode, the counter electrode was platinum plate, and the electrolyte was 3.5% NaCl solution. The scan rate of potentiodynamic polarization experiments was 2 mV/s. The potential scans were started from −1200 mV to 0 mV [18]. Before the electrochemical impedance spectroscopy (EIS) measurements, the specimens were immersed into the 3.5% NaCl solution for 5 h, in order to achieve a steady state for the specimen surface. The measuring frequency range was from 0.01 Hz to 10^5^ Hz with perturbation voltage amplitude of 0.01 mV. The obtained EIS data were analyzed by ZSimpWin software Version 3.30 [19].

The samples after solution treatment were prepared according to standard metallographic techniques and observed by ZEISS M10A SEM equipped with X-ray EDS system. To verify the consistency of the result, five SEM photos where at least 10 particles were analyzed by EDS. The intragranular precipitates and the grain boundary precipitates were observed by Tecnai G2 20 ST transmission electron microscope (TEM, Thermo Fisher Scientific, Waltham, MA, USA) equipped with X-ray EDS system, operated at 200kV, and the diffraction direction was [001]_Al_. The initial samples were ground on 80, 600, 1500, and 2000 grit SiC paper to a thin layer of around 100 µm in thickness. Disks of 3mm in diameter were stamped out from the film. Then the TEM samples were prepared by twin-jet electropolishing method with electrolyte made of 70% methanol and 30% nitric acid at temperature of −25 °C. Quantitative TEM analysis was conducted on five positions of samples, where at least 100 particles were counted and measured to determine their sizes by Image J software. The number density has been estimated by dividing the number of precipitates by the volume (thickness of the foil × area) of the field. The volume fraction of precipitates has been estimated by multiplying the number density and the average volume of the precipitates.

## 3. Results

### 3.1. Microstructure

**Figure 1:**
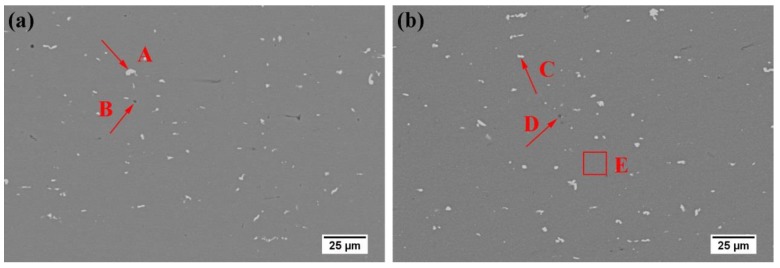
Scanning electron microscope (SEM) morphologies of alloy AA (**a**) and AZ (**b**) after solution treatment.

Figure 1 shows the SEM morphology of alloy AA and AZ after quenching. There are two types of secondary phase particles that can be observed in the alloy matrix: one is a white particle, which is mostly in strips and blocks and uniformly distributed in the matrix, the other is a black or grey spheroidal phase. EDS analysis results of Table 2 show that the white particles are Al-Fe-Mn-Si phase, and the black or grey particles are Mg_2_Si phase [20]. Image J software was used to analyze multiple fields of view of alloys AA and AZ, and the area fractions of the second phase particles were 1.29% and 1.24%, respectively. It can be seen that the addition of Zn has little effect on the secondary phase particles after quenching. However, we can find that the Zn atoms dissolved into matrix, according to the EDS results of the red square E, as shown in Table 2.

**Figure 2:**
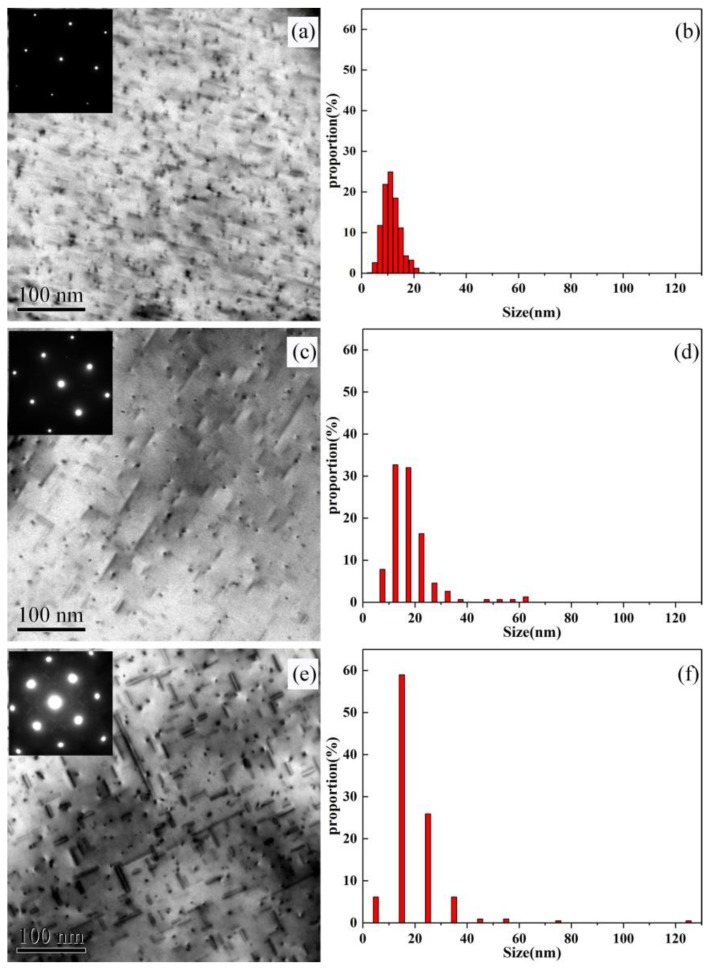
Transmission electron microscope (TEM) on intragranular precipitates of alloy AA and the size distribution of needle phase under 170 °C aging (**a**,**b**) under aged state, (**c**,**d**) peak aged state, and (**e**,**f**) over aged state.

**Figure 3:**
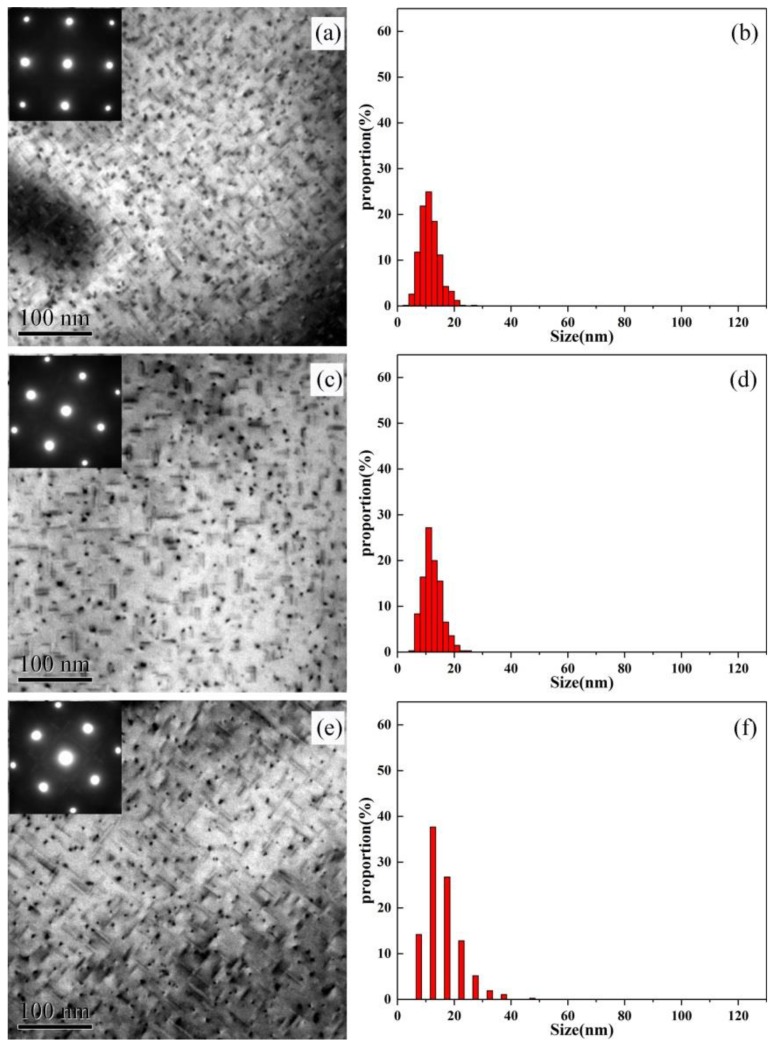
TEM on intragranular precipitates of alloy AZ and the size distribution of needle phase under 170 °C aging (**a**,**b**) under aged state, (**c**,**d**) peak aged state, and (**e**,**f**) over aged state.

The TEM images of intragranular precipitates and the size distributions of needle-shaped phases of the alloys AA and AZ, which were artificial aged at 170 °C are shown in Figure 2 and Figure 3. It can be seen from Figure 2 and Figure 3 that the precipitates of alloy AA and AZ are mainly needle-shaped and point-shaped, according to the diffraction pattern show in Figure 2 and Figure 3, the two shaped precipitates both being Mg-Si phases [21,22], and η-MgZn_2_ precipitates were not observed. According to the histograms of size distributions shown in Figure 2 and Figure 3, it is obvious that, with the increase of aging time, the precipitates become coarser, and the proportion of the large precipitates also increase, especially for alloy AZ with Zn addition. According to the quantitative TEM results displayed in Table 3, we can see that the length and the volume fraction increase as the aging time increases, leading the distance between precipitates (λ) to become shorter. The number density decreased due to the coarsening of the precipitates after over aging. For alloy AZ with the addition of Zn, the number density and the volume fraction are both higher than for alloy AA, while the length of the needle-shaped phase was lower than for alloy AA. This means that the addition of Zn was beneficial to obtain finer and denser intragranular structure. In addition, at the over-age state, there were significantly larger precipitates in alloy AA with a size of about 130 nm, while the precipitates in alloy AZ had a size of about 40 nm. It can be concluded that the addition of Zn has a significant effect on the coarsening of the precipitates. 

**Figure 4:**
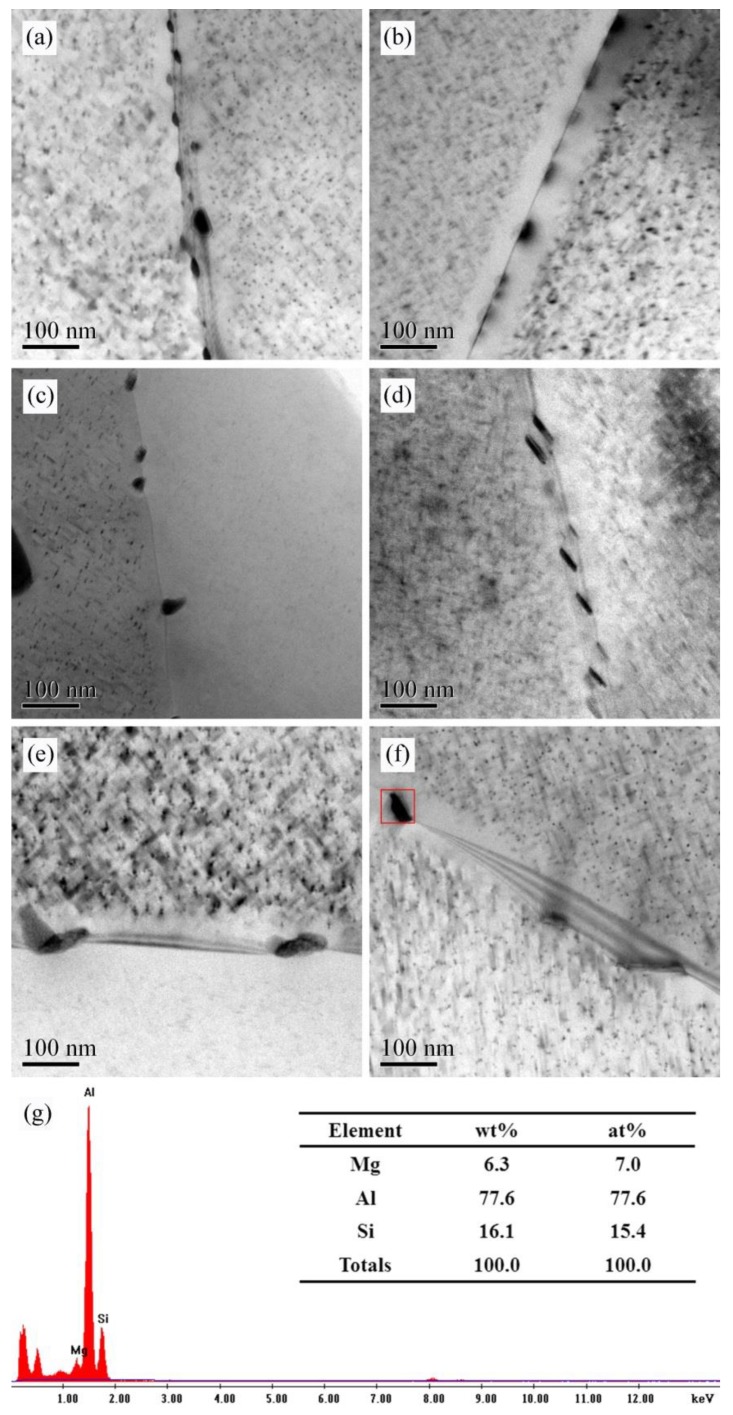
TEM and EDS analysis on grain boundary precipitates of alloy AA and AZ under aging at 170 °C: (**a**,**c**,**e**) alloy AA, (**b**,**d**,**f**) alloy AZ, (**a**,**b**) under aged state, (**c**,**d**) peak aged state, (**e**,**f**) over aged state, and (**g**) EDS analysis of the precipitates.

As Figure 4 shows, both the AA and AZ alloys have grain boundary phase precipitation after 4 h of aging. In addition, the size of grain boundary precipitates increased with the aging time, but the number of precipitates decreased. Comparing the size of grain boundary precipitates of alloy AA and AZ under the same aging state, the grain boundary precipitates of alloy AZ were slightly larger than those of alloy AA. Figure 4g shows the EDS analysis results of the precipitate in the red square of Figure 4f. It is shown that the grain boundary precipitates do not contain Zn for the alloy AZ with 0.2% Zn added. Although the addition of 0.2% Zn will have some influence on the size and morphology of the precipitates at the grain boundaries, the influence on the composition of the precipitates needs to be further studied.

**Figure 5:**
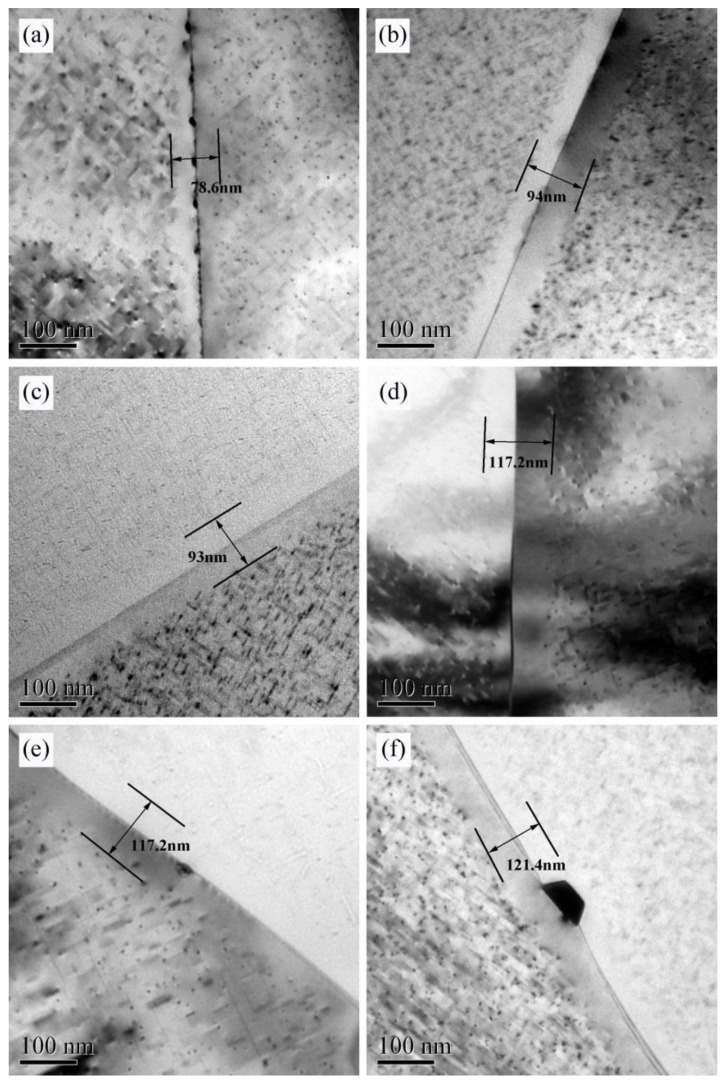
TEM on PFZ of alloy AA and AZ under aging at 170 °C: (**a**,**c**,**e**) alloy AA, (**b**,**d**,**f**) alloy AZ, (**a**,**b**) under aged state, (**c**,**d**) peak aged state, and (**e**,**f**) over aged state.

Figure 5 shows the PFZ of alloys AA and AZ under different aging conditions. According to the measured width of the PFZ, we found that the PFZ of alloy AA and AZ all became wider with the increase of aging time. Moreover, compared to alloy AA, the PFZ of alloy AZ was broader. However, for the alloy AZ, we can observe from Figure 5d,f that the width of PFZ is small, only increasing by 4.2 nm after reaching the over aged condition. This is due to the fact that the density of precipitates for alloy AZ was higher than for alloy AA (see Table 3), and more solute atoms were consumed during the growth of precipitates after peak aging, which hindered the segregation of solute atoms along grain boundary, thereby reducing the probability of PFZ broadening [23].

### 3.2. Hardness and Tensile Properties

**Figure 6:**
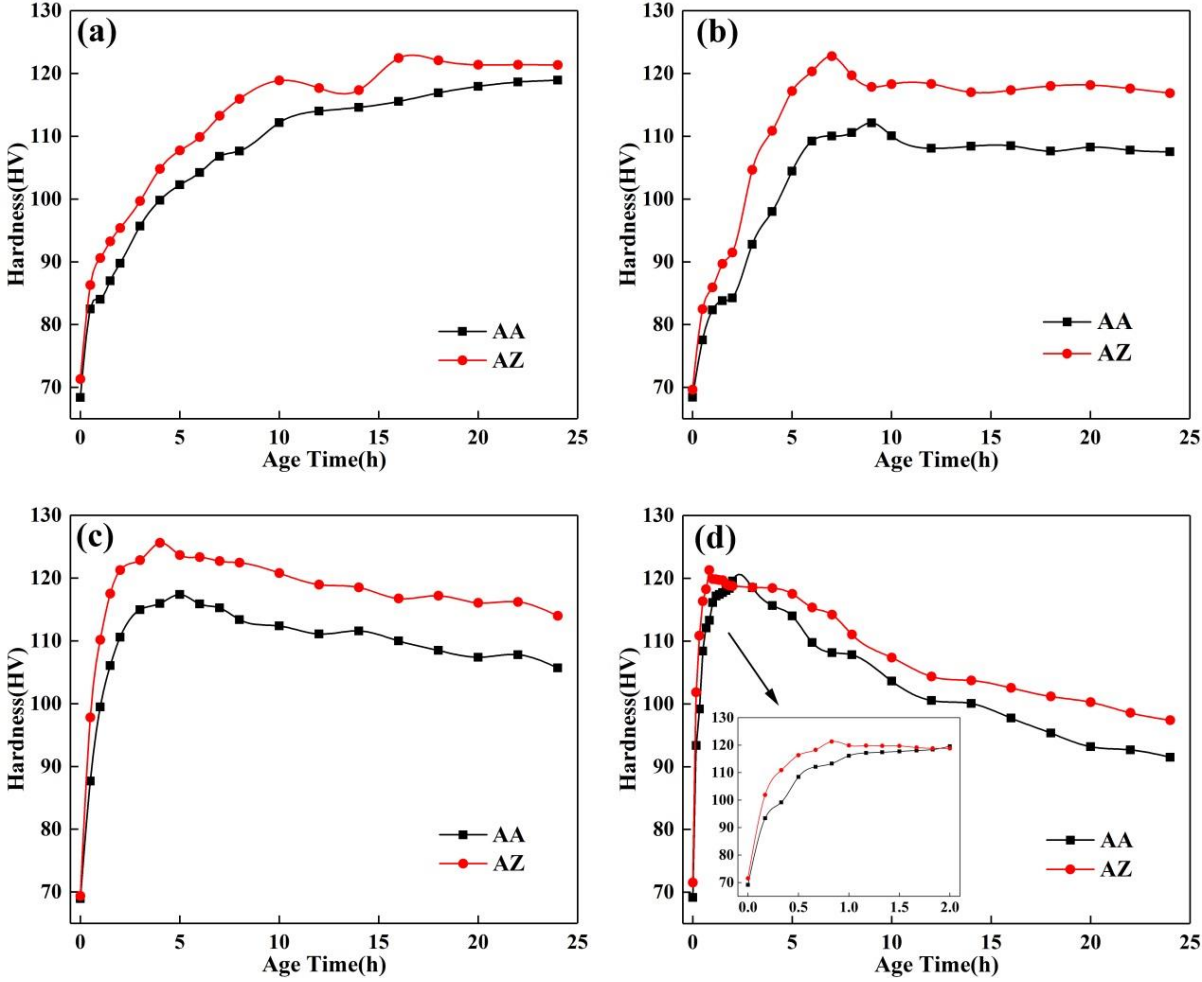
Age hardening curves of the alloy AA and AZ at different temperatures: (**a**) 150 °C, (**b**) 170 °C, (**c**) 190 °C, and (**d**) 210 °C.

Figure 6 shows the age hardening curves of the alloys AA and AZ at different temperatures, and the aging time and hardness of alloys AA and AZ under peak aging conditions at different temperatures are shown in Table 4. According to the hardness of the quenched alloy at different aging temperatures, it seems that the 0.2% Zn addition does not significantly influence the solution strengthening effect, which was corresponded to the SEM morphologies as shown in Figure 1. Figure 6a shows the age hardening curves of alloys AA and AZ at 150 °C. The peak aging occurs first for alloy AZ at 16 h, for which the hardness is 122 ± 1.9 HV, and occurs at 24 h for alloy AA, for which the hardness is 119 ± 0.3 HV. Figure 6b shows the age hardening curves of alloys AA and AZ at 170 °C, the peak aged state of alloy AZ reached at 7 h, and the hardness was 123 ± 1.1 HV, while the peak aged hardness of alloy AA was112±1.4 HV, when the aging time was 9 h. Figure 6c displays the age hardening behavior of alloys AA and AZ at 190 °C. When aged for 4 h, alloy AZ reached peak aged state, with a hardness of 126 ± 0.9 HV. For the alloy AA, the peak aging time was 5 h, with a hardness of 117 ± 0.8 HV. Figure 6d shows that the peak aged state at 210 °C was reached after 2 h for alloy AA, with a hardness of 120 ± 0.4 HV, and after 50 min for alloy AZ with a hardness of 121 ± 1.1 HV. The obtained results on age hardening phenomena at different temperatures indicate that the addition of 0.2% Zn can accelerate the age hardening response, and enhance the hardness of Al-Mg-Si alloy. This can be further confirmed by inset picture of Figure 6d: after aging for 20 min, the hardness increments of alloys AA and AZ were 30 HV and 40 HV. 

**Figure 7:**
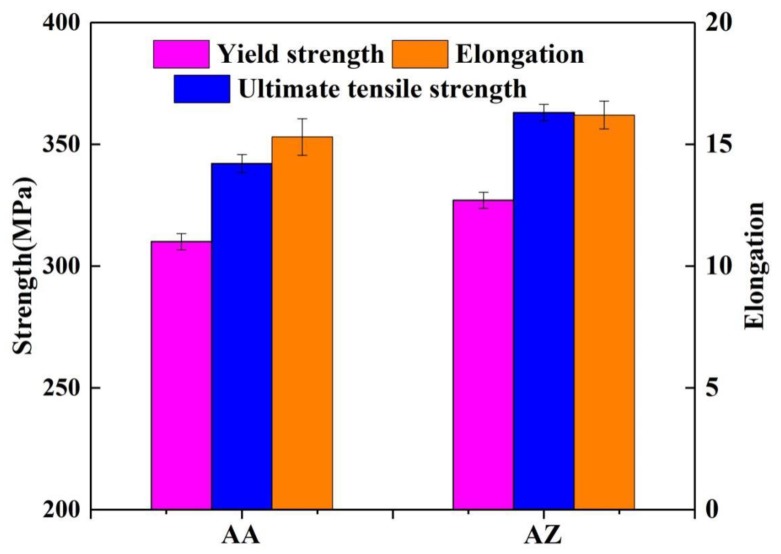
Tensile test results of alloy AA and AZ under peak aged conditions at 170 °C.

**Figure 8:**
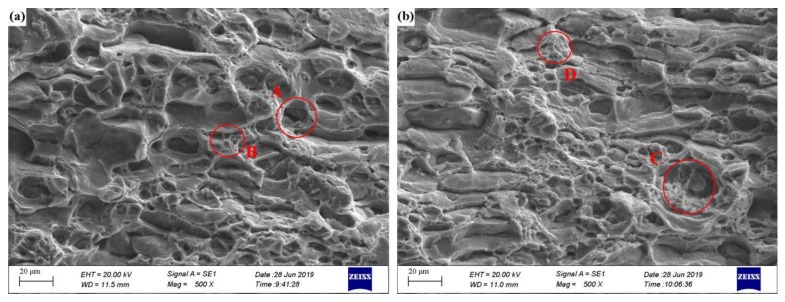
SEM fractography of alloys AA (**a**) and AZ (**b**) under peak aged state at 170 °C.

The tensile test results of the two alloys under peak aged conditions at 170 °C are shown in Figure 7. The detailed mechanical properties are shown in Table 5. The Zn containing alloy AZ exhibited good mechanical properties, with yield strength (YS) and ultimate tensile strength (UTS) of 327 and 363 MPa, respectively, which were higher than for alloy AA (310 and 342 MPa). The elongation of alloy AA and AZ was 15.3% and 16.2%, respectively. The measured tensile properties revealed that the Zn addition can improve the YS and UTS, which was well correlated with the results shown in Figure 6b. Figure 8 shows the SEM fractography of alloys AA and AZ under peak aged state at 170 °C. Both alloy AA and AZ all show typical ductile fracture with the large-size dimples (like the red circles A and C shown in Figure 8) and small-size dimples (like the red circles B and D shown in Figure 8). The area fraction of the transgranular fracture (A_At_) was 0.319 for alloy AZ, a little higher than for alloy AA (0.309), while the area fraction of the coarse voiding (A_Ap_) was 0.160 for alloy AZ, lower than for alloy AA (0.202). It is worth noting that alloy AZ showed better plasticity (higher elongation at fracture) than alloy AA, which was demonstrated by the tensile tests. 

### 3.3. IGC Properties

**Figure 9:**
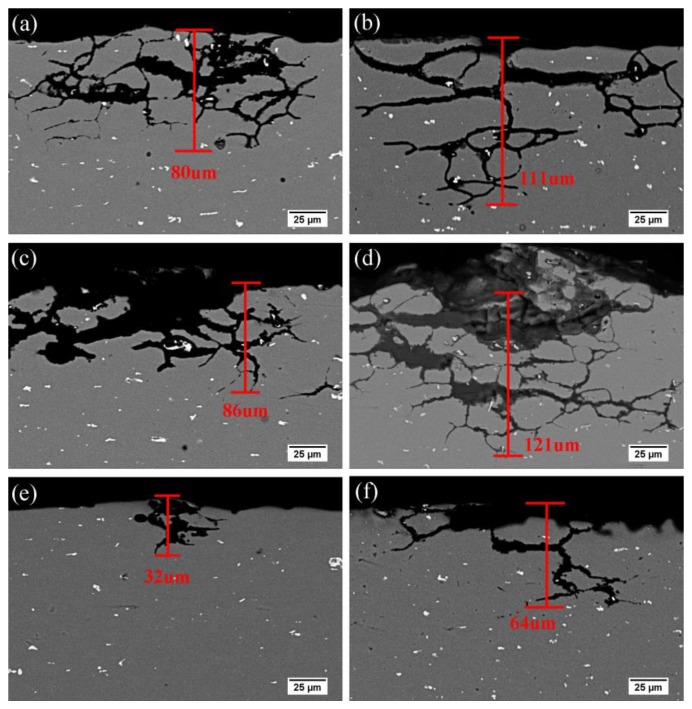
SEM morphologies of IGC and the maximum corrosion depth of alloy AA and AZ under different aging conditions at 170 °C (**a**,**c**,**e**) alloy AA, (**b**,**d**,**f**) alloy AZ, (**a**,**b**) under aged state, (**c**,**d**) peak aged state, and (**e**,**f**) over aged state.

In order to verify the effect of Zn addition on the corrosion properties of alloys, the immersion tests were made. The obtained IGC morphology and the maximum corrosion depths of the alloys are shown in Figure 9. At peak aged state, alloy AA and AZ showed the worst corrosion property, and the maximum corrosion depths were 86 μm and 121 μm, respectively. The corrosion resistance of the under aged alloys was higher than that in peak aged state, and the depths were 80 μm and 111 μm, respectively. The over aged alloys displayed the best corrosion resistance: the maximum corrosion depths of alloy AA and AZ were 32 and 64 μm, respectively. In addition, at the same aged condition, the alloy AZ with 0.2 wt.% Zn addition showed more sensitivity to IGC attack. It can be concluded that the addition of Zn and different aging conditions both play an important role on the IGC susceptibility. The reasons may be related to the different precipitate distribution characteristics, which will be discussed later on.

### 3.4. Electrochemical Results

**Figure 10:**
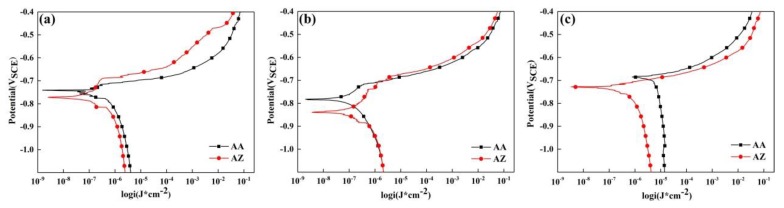
Potentiodynamic polarization curves of alloy AA and AZ under different aging conditions at 170 °C: (**a**) under aged, (**b**) peak aged, and (**c**) over aged.

Corrosion potential was a signal to reflect the corrosion tendency of the alloy: the more positive is E_corr_, the lower the tendency of the alloy to experience electrochemical corrosion [24]. The corrosion current density presents the electrochemical corrosion rate of the alloy: the larger the value of I_corr_, the faster the corrosion rate, and the poorer the corrosion resistance of the alloy. Figure 10 showed the potentiodynamic polarization curves of alloy AA and AZ under different aging conditions. E_corr_ and I_corr_ calculated by Tafel method are shown in Table 6. It can be seen that E_corr_ tends to be more negative at under aged and peak aged conditions of alloy AA and AZ. Furthermore, the E_corr_ value was most negative and the I_corr_ value was highest at peak aged state. For over aged condition, the E_corr_ of alloys AA and AZ shifted to be positive, and the I_corr_ was smallest. This means that the over aged alloys AA and AZ can obtain the best corrosion properties, whilst, the E_corr_ turned to negative values and the I_corr_ increased with the addition of 0.2% Zn. The result of the potentiodynamic polarization curves were totally matched to the immersion tests exhibited in Figure 9.

**Figure 11:**
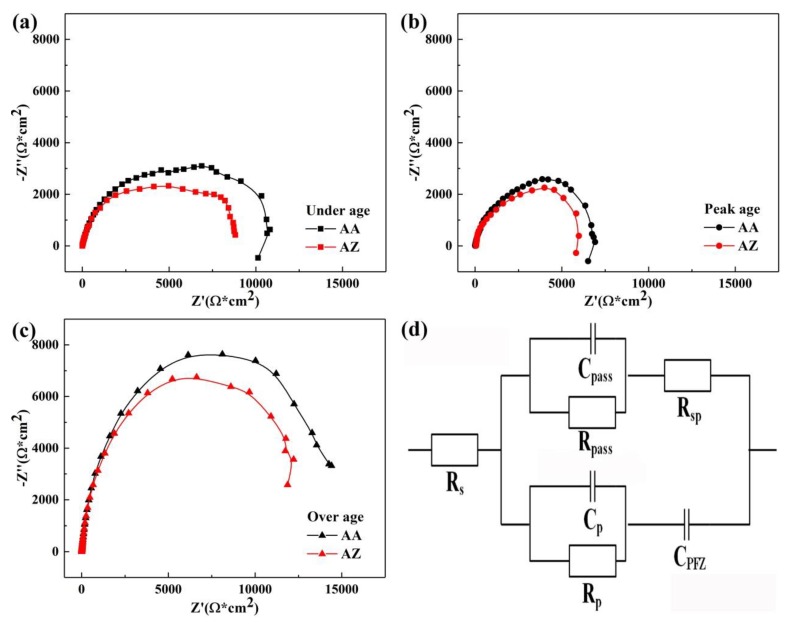
Nyquist plots of alloy AA and AZ under different aged conditions at 170 °C: (**a**) under aged, (**b**) peak aged, (**c**) over aged, and (**d**) equivalent circuit.

The Nyquist plots of alloy AA and AZ at different aged conditions are shown in Figure 11. The equivalent circuit for EIS test is shown in Figure 11d. The physical meaning of the circuit elements is described as follows: R_s_ is the resistance of the electrolyte, R_pass_ is the resistance of the oxide film, C_pass_ is the capacitance of the oxide film, R_sp_ is the resistance of the second phases R_p_ is the ohmic resistance of the precipitates, C_p_ is the capacitance of the precipitates and C_PFZ_ is the capacitance of the PFZ. The parameters of EIS are shown in Table 7. We can roughly derive that the corrosion susceptibility of alloy AZ was higher than that of alloy AA, due to the fact that the diameter of the capacitive reactance arcs of alloy AA were all larger than for alloy AZ at the different aging states. The diameter of capacitive reactance arcs was largest for the over aged state, and was lowest for the peak aged state both for alloys AA and AZ, leading to the best corrosion properties at over aged state, and the worst corrosion properties at peak aged state. Furthermore, according to the literature [18], we know that a system with lower capacitances and higher resistances (ignore R_s_) can obtain better corrosion resistance. We can find that the resistance of alloy AZ with 0.2% Zn addition was lower and the capacitance was higher than for alloy AA. Further analysis of the parameters shown in Table 7 will be carried out later on.

## 4. Discussion

### 4.1. Effect of Zn Addition of Precipitation Behavior

The intragranular precipitates morphology shown in Figure 2 and Figure 3 indicated that the minor addition of Zn was benefit for alloys to get finer and denser intragranular precipitates, while the MgZn precipitates were not observed. It is because the minor addition of Zn did not change the precipitation sequence of Al-Mg-Si alloys that the precipitates of alloy AZ still were Mg_2_Si phases. According to the result of Figure 2a and Figure 3a, we can find that at the under aged condition, the alloy AZ with Zn addition obtained higher density of GP zones than alloy AA, and more GP zones evolved into β″ phase. It follows that the addition of Zn can improve the solid solubility of alloy AZ, so that the formation of GP zones can be facilitated, providing more nucleation sites for β″ phase, and accelerating the transformation of GP zones to β″ phase. This opinion was similar to Zhu′s study [25]: he considered that the addition of Zn in Al-Mg-Si-Cu alloy can accelerate partitioning of Mg, Si, and Cu atoms from Al matrix into solute aggregates, which can promote the formation of clusters, and facilitate the clusters to convert into GP zones, β″ phase. It was shown in Table 3 that the number of precipitates for alloy AZ was higher than for alloy AA, and the size was finer, although alloy AZ shows faster age hardening response, and can reach the peak aged state more quickly; however, after aging for 24 h, the precipitates of alloy AA were still coarser than those of alloy AZ (as shown in Figure 2e and Figure 3e). It is declared that the addition of Zn can enhance the precipitation strengthening effect. This is the reason why the hardness of alloy AZ was always higher than that of alloy AA. 

The Johnson–Mehl–Avrami (JMA) equation was applied to further analyze the precipitation kinetics for the alloys. The equation is [26]

(1) $$f\left(t\right)=1-\exp\left[-{\left(\mathrm{Kt}\right)}^{n}\right]$$

where K is the rate constant, n is the Avrami exponent, which reflects the nucleation and growth mechanisms, t is the aging time, and f(t) is the transformed fraction which can be evaluated by the change of physical properties changed with time. For this study, the f(t) was calculated by the hardness [27]

(2) $$f\left(t\right)=\frac{H_{t}-H_{0}}{H_{m}-H_{0}}$$

where *H_0_* represents the hardness after water quenching, *H_t_* means the hardness at aging time (t), *H_m_* is the maximum hardness during artificial aging.

In order to obtain the linearization of Equation (1), we get the equation

(3) ln(ln(1/1−f))=nlnt+nlnK

**Figure 12:**
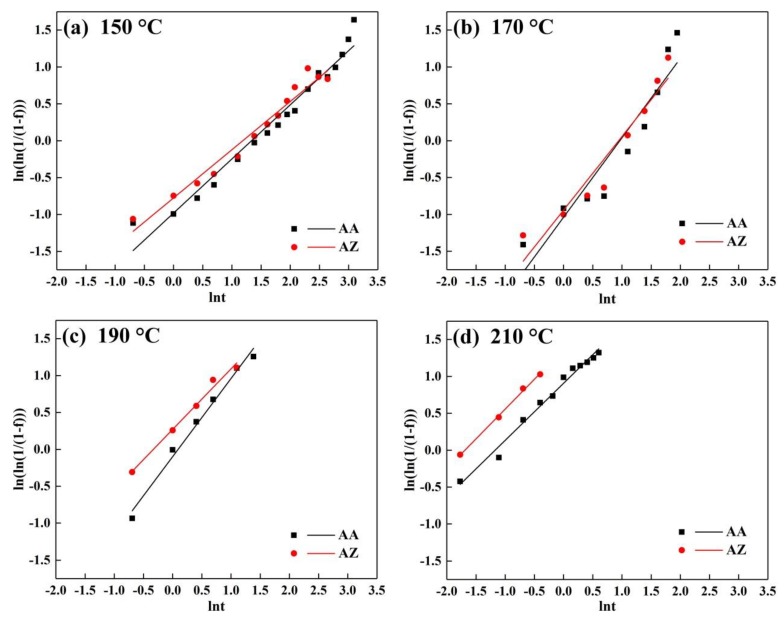
Johnson–Mehl–Avrami (JMA) plots of alloy AA and AZ at different aging temperatures: (**a**) 150 °C, (**b**) 170 °C, (**c**) 190 °C, and (**d**) 210 °C.

The results are shown in Figure 12: the slope and y-intercept represent n and nlnK, respectively, and the values are shown in Table 8. The values of the Avrami exponent (n) fluctuate around 0.8. This means that the precipitation mechanisms did not change with the Zn addition at different aging temperatures. The reaction rate constant K was increased with the aging temperature for alloy AA and AZ. Especially, K of the alloy AZ was higher than for the alloy AA at any aging temperature. It is suggested that the precipitation kinetics not only increase with the aging temperature, but also with the addition of Zn, which is in accordance with the intragranular precipitates shown in Figure 2 and Figure 3.

### 4.2. Correlation of Precipitation Behavior and Strength

The precipitation strengthening of the alloys was strongly related to the type, morphology, size and distribution of the precipitates [28]. On account of the Zn addition the precipitation sequence did not change, and for the β″ phases, the strengthening increment can be described as [29]

(4) $$\tau_{c}=\frac{Gb}{2\pi \sqrt{\left(1-v\right)}}\left\{\frac{1}{\left(1.075\sqrt{\frac{0.433\pi }{f_{v}}}-\sqrt{1.732}\right)}\right\}\ln\frac{\sqrt{1.732}D}{r_{0}}$$

where τ_c_ is the critical resolved shear stress (CRSS) increment which was correlated to the precipitation strengthening, G is the shear modulus of the Al matrix, b is the Burgers vector of the dislocations and v the Poisson ratio, f_v_ is the volume fraction of the intragranular precipitates, D is the diameter of the needle phase, r_0_ is the cut-off radius around the dislocations. The value of parameters of Equation (4) and τ_c_ are shown in Table 9, according to the Equation (1), inferring that the volume fraction and the diameter of the intragranular precipitates were the main parameters affecting the CRSS. Meanwhile, the alloy AZ with 0.2% Zn addition got higher precipitation strengthening effect as a result of higher volume fraction and similar diameter of the precipitate, and the calculated results agreed totally with the tensile test results.

The fracture toughness represents the ability of alloys to prevent crack growth, which is influenced by the microstructural features such as the intermetallic (IM) phase, intragranular precipitates, dispersoids, PFZ, grain size and so on. The equation to calculate the fracture toughness K_Ic_ is [30]

(5) $$K_{\mathrm{Ic}}={\left[2\cdot \sigma_{y}\cdot E\cdot {\left(\frac{\pi }{6}\right)}^{1/3}\cdot l\right]}^{1/2}\cdot f_{\nu }{}^{-\frac{1}{6}}\cdot A_{\mathrm{Ap}}{}^{-m}\times \exp\left[{\left(A_{\mathrm{At}}\right)}^{1/2}\cdot \exp\left(-{\left(\frac{N\cdot W_{\mathrm{PFZs}}}{\delta }\right)}^{1/2}\right)\right]$$

where σ_y_ is the yield strength obtained by the tensile experiment, E is the Young′s modulus, l is the average length of the coarse particles, f_v_ is the volume fraction of the IM phase, A_Ap_ is the area fraction of the coarse voiding counted from Figure 8, A_At_ is the area fraction of the transgranular fracture, W_PFZs_ is the PFZ width of alloy AA and AZ, *δ* is the mean distance between the coarse IM particles, m is connected with the volume fraction of the coarse IM phase (for lower fraction m is similar to 0.3, for higher fraction m is similar to 0.5), N is a number of PFZs that were distributed between the coarse IM particles at distance of *δ*. There, the N is equal to 3. The values of the parameters of Equation (5) and the calculation result of K_Ic_ are shown in Table 10. The alloy AZ showed better fracture toughness (K_Ic_) than the alloy AA. This indicates that the Zn addition improves the strength of the alloys, while the fracture toughness did not decrease. This is coherent with the elongation at fracture of alloys AA and AZ.

### 4.3. Correlation of Precipitation Behavior and IGC

It can be discovered from Figure 9 that intergranular corrosion of the alloys happened surrounded in the surroundings of the white Al-Fe-Mn-Si phases. At the same time, the morphology and distribution of the coarse Fe-rich phase shown in Figure 1 did not have significant differences, and the R_sp_ shown in Table 6 was relatively smaller than R_p_, R_pass_. Therefore, in this investigation, the characteristics of the white phase were not the major factor leading to the difference in IGC properties between alloys AA and AZ. Many studies have shown that the IGC sensitivity of Al-Mg-Si alloys is correlated to the grain boundary precipitates and PFZ. The disparity of the electrochemical microcouples, which combined the matrix with the PFZ and the grain boundary precipitates with the PFZ, was mainly responsible for the difference of the IGC sensitivity [7]. The research of Eckermann et al. [31] revealed that, at the beginning of the corrosion, the MgSi particles quickly dissolved as an effective cathode, especially when the particles were distributed in the grain boundary. As can be seen from Figure 4, at under aged state and peak aged state, the number density of grain boundary precipitates of alloy AA and AZ was higher, and the size was smaller. This contributed to form the electrochemical microcouples between the grain boundary precipitates and the PFZ, and lead the alloys to be more prone to IGC. Furthermore, the PFZ width of alloy AA and AZ at peak aged state was broader than that at under aged state, as shown in Figure 5. Therefore, the IGC susceptibility of the alloys at peak aged state was higher. For the over aged condition, the PFZ width was largest than ever. However, the solute concentrations of Mg atoms and Si atoms in the matrix were lower due to the growth of the intragranular precipitates, making the corrosion potential between the matrix and PFZ to get closer. Moreover, with the grain boundary precipitates becoming coarser, and the distance between the precipitates becoming larger, it is hard to form continued electrochemical microcouples, thus increasing the corrosion resistance of the alloys. The results in Figure 4 and Figure 5 showed that the grain boundary precipitates of alloy AZ were larger than those of alloy AA, and the PFZ width was also larger than for alloy AA, so the IGC properties of alloy AZ were weaker than for alloy AA. 

It can be also proved by the result, shown in Table 6, that R_p_ increased with the aging time, because of the increase of volume fraction of the precipitates. In the meantime, the decrease of the distance between the precipitates as shown in Table 3 makes the increase of the C_p_ due to capacitance to be negatively associated with distance, so that the R_p_ of alloy AZ was higher than that of alloy AA, and C_p_ was lower than that of alloy AA. We find that C_PFZ_ increases first and decrease as the aging time increases, and the C_PFZ_ of alloy AZ was all higher than that of alloy AA, although the increase of aging time and the Zn addition make the width of the PFZ to increase. It may be attributed to the complex relationship between the PFZ and the grain boundary precipitates. R_pass_ was higher than other parameters: the R_pass_ of alloy AA was 15,480 Ω·cm^2^ at over aged state, which was higher than 13,750 Ω·cm^2^ for alloy AZ. From the above-mentioned, we can conclude that the corrosion resistance of alloy AA was higher than that of alloy AZ, and that the peak aged alloy was much more sensitive to corrosion, the over aged alloy shows greatest corrosion properties with a higher R_corr_ and lower capacitance.

## 5. Conclusions

1. The addition of Zn does not change the precipitation sequence of Al-Mg-Si alloys, but increases the precipitation kinetics. Moreover, the addition of Zn is beneficial to obtain finer and denser intragranular precipitates, and can suppress the coarser of intragranular precipitates during over aged condition.
2. The addition of Zn accelerated the age hardening response of the alloys and improved the hardness. The age hardening response was faster with increasing aging temperature. The Zn addition can enhance the tensile properties without decreasing the fracture toughness.
3. As the addition of Zn increases the width of PFZ, and the corrosion potential between PFZ and the matrix becomes larger, the corrosion resistance decreases. The alloy shows best corrosion resistance under over aged state, and worst corrosion resistance under peak aged state.

## Figures and Tables

**Table 1:** Chemical composition of AA and AZ alloys (wt.%).

Alloy	Si	Fe	Zn	Mg	Mn	Ti	Al
AA	1.08	0.15	0.05	0.85	0.59	0.02	Bal
AZ	0.97	0.10	0.20	0.91	0.56	0.01	Bal

**Table 2:** Energy dispersive spectrometer (EDS) results of alloy AA and AZ after solution treatment (at %).

Spots	Al	Mg	Si	Fe	Mn	Zn
A	77.8	/	8.2	8.2	5.8	/
B	98.2	0.7	1.1	/	/	/
C	93.8	/	3.4	1.0	1.8	/
D	97.9	0.9	1.2	/	/	/
E	97.6	0.9	1.4	/	/	0.1

**Table 3:** Quantitative TEM studies of precipitates in the alloys AA and AZ.

Precipitates’ Size and Distribution	Alloy AA			Alloy AZ		
	Under Aged	Peak Aged	Over Aged	Under Aged	Peak Aged	Over Aged
Precipitate length (nm)	15.6	17.7	19.1	10.9	11.7	15.4
Number density × 10^4^ (μm^−3^)	7.35	5.76	5.34	11.79	8.15	7.50
Volume fraction (%)	0.36	0.87	1.35	0.40	1.35	1.92
λ (nm)	14.29	11.93	9	10.6	8.7	7.84

**Table 4:** Aging time and hardness of alloys AA and AZ under peak aging conditions at different temperatures.

T (°C)	150		170		190		210	
Alloy	AA	AZ	AA	AZ	AA	AZ	AA	AZ
Time (h)	24	16	9	7	5	4	2	0.8
Hardness (HV)	119 ± 0.3	122 ± 1.9	112 ± 1.4	123 ± 1.1	117 ± 0.8	126 ± 0.9	120 ± 0.4	121 ± 1.1

**Table 5:** Mechanical properties of alloy AA and AZ under peak aged conditions at 170 °C.

Alloy	YS (MPa)	UTS (MPa)	Elongation (%)
AA	310	342	15.3
AZ	327	363	16.2

**Table 6:** E_corr_, I_corr_ of alloy AA and AZ under different aging conditions at 170 °C.

Alloy	Under Aged		Peak Aged		Over Aged	
	E_corr_ (V_SCE_)	I_corr_ (μA/cm^2^)	E_corr_ (V_SCE_)	I_corr_ (μA/cm^2^)	E_corr_ (V_SCE_)	I_corr_ (μA/cm^2^)
AA	−0.741	0.154	−0.782	0.199	−0.685	0.123
AZ	−0.772	0.482	−0.84	0.595	−0.728	0.424

**Table 7:** Parameters of EIS test.

Parameters	Alloy AA			Alloy AZ		
	Under Aged	Peak Aged	Over Aged	Under Aged	Peak Aged	Over Aged
R_s_ (Ω·cm^2^)	0.8288	4.774	3.555	3.475	5.791	1.182
R_pass_ (Ω·cm^2^)	4300	3939	15480	3356	3058	13750
C_pass_ (μF·cm^2^)	2.265	2.835	4.8	5.908	9.896	6.086
R_sp_ (Ω·cm^2^)	212.5	135.7	145.4	190.98	277	316.6
R_p_ (Ω·cm^2^)	1368	1869	2479	1730	2039	3830
C_p_ (μF·cm^2^)	1.082	1.087	1.424	1.103	1.123	1.572
C_PFZ_ (μF·cm^2^)	2.16	2.607	1.624	4.154	4.786	1.675

**Table 8:** Values of n and K for alloy AA and AZ at different aging temperatures.

T (°C)	150		170		190		210	
Alloy	AA	AZ	AA	AZ	AA	AZ	AA	AZ
n	0.73	0.71	1.08	1.00	0.97	0.82	0.74	0.81
K (s^−1^)	0.26	0.30	0.38	0.39	0.98	1.39	3.23	5.42

**Table 9:** Value of parameters of Equation (4) and τ_c_ of alloy AA and AZ at peak aged state.

Alloy	G (GPa)	b (nm)	v	f_v_ (vol %)	D (nm)	r_0_ (nm)	τ_c_ (MPa)
AA	28	0.286	0.3	0.87	3.3	0.572	50.93
AZ	28	0.286	0.3	1.35	4.4	0.572	74.43

**Table 10:** Values of parameters of Equation (5) and KIc of alloys AA and AZ at peak aged state.

Alloy	σ_y_ (MPa)	E (GPa)	l (µm)	f_v_ (vol %)	δ (µm)	W_PFZs_ (µm)	A_Ap_	A_At_	K_Ic_ (MPa·m^1/2^)
AA	310	69	2.53	1.29	10.69	0.093	0.202	0.309	23.21
AZ	327	69	2.47	1.24	14.81	0.117	0.160	0.319	25.50

## References

[1] Guo M.X., Zhang Y.D., Li G.J., Jin S.B., Sha G., Zhang J.S., Zhuang L.Z., Lavernia E.J. (2019). Solute clustering in Al-Mg-Si-Cu-(Zn) alloys during aging. J. Alloy Compd..

[2] Yan L., Zhang Y., Li X., Li Z., Wang F., Liu H., Xiong B. (2014). Effect of Zn addition on microstructure and mechanical properties of an Al–Mg–Si alloy. Prog. Nat. Sci. Mater. Int..

[3] Guo M.X., Sha G., Cao L.Y., Liu W.Q., Zhang J.S., Zhuang L.Z. (2015). Enhanced bake-hardening response of an Al–Mg–Si–Cu alloy with Zn addition. Mater. Chem. Phys..

[4] Zhi-xiu W., Hai L., Jian-hua G., Ren-guo S., Zi-qiao Z. (2012). Effect of Cu content on microstructures andproperties of Al-Mg-Si-Cu alloys. Chin. J. Nonferr. Metal.

[5] Zou Y., Liu Q., Jia Z., Xing Y., Ding L., Wang X. (2017). The intergranular corrosion behavior of 6000-series alloys with different Mg/Si and Cu content. Appl. Surf. Sci..

[6] El-Menshawy K., El-Sayed A.-W.A., El-Bedawy M.E., Ahmed H.A., El-Raghy S.M. (2012). Effect of aging time at low aging temperatures on the corrosion of aluminum alloy 6061. Corros. Sci..

[7] Li H., Zhao P., Wang Z., Mao Q., Fang B., Song R., Zheng Z. (2016). The intergranular corrosion susceptibility of a heavily overaged Al-Mg-Si-Cu alloy. Corros. Sci..

[8] Wang Z., Li H., Miao F., Sun W., Fang B., Song R., Zheng Z. (2014). Improving the intergranular corrosion resistance of Al–Mg–Si–Cu alloys without strength loss by a two-step aging treatment. Mater. Sci. Eng. A.

[9] Berg L.K., Gjϕnnes J., Hansen V., Li X.Z., Knutson-Wedel M., Waterloo G., Schryvers D., Wallenberg L.R. (2001). GP-zones in Al–Zn–Mg alloys and their role in artificial aging. Acta Mater..

[10] Saito T., Ehlers F.J.H., Lefebvre W., Hernandez-Maldonado D., Bjørge R., Marioara C.D., Andersen S.J., Holmestad R. (2014). HAADF-STEM and DFT investigations of the Zn-containing β″ phase in Al–Mg–Si alloys. Acta Mater..

[11] Pogatscher S., Antrekowitsch H., Leitner H., Ebner T., Uggowitzer P.J. (2011). Mechanisms controlling the artificial aging of Al–Mg–Si Alloys. Acta Mater..

[12] Cai Y.H., Wang C., Zhang J.S. (2013). Microstructural characteristics and aging response of Zn-containing Al-Mg-Si-Cu alloy. Int. J. Miner. Metall. Mater..

[13] Ding X.P., Cui H., Zhang J.X., Li H.X., Guo M.X., Lin Z., Zhuang L.Z., Zhang J.S. (2015). The effect of Zn on the age hardening response in an Al–Mg–Si alloy. Mater. Design.

[14] Guo M.X., Zhang Y., Zhang X.K., Zhang J.S., Zhuang L.Z. (2016). Non-isothermal precipitation behaviors of Al-Mg-Si-Cu alloys with different Zn contents. Mater. Sci. Eng. A.

[15] Xu C., He H., Yu W., Li L. (2019). Influence of quenching temperature on peak aging time and hardness of Al-Mg-Si-Cu alloys strengthened by nano-sized precipitates. Mater. Sci. Eng. A.

[16] GB/T 228.1-2010 (2010). Metallic materials-Tensile testing-Part 1: Method of test at room temperature.

[17] GB/T 7998-2005 (2005). Test method for intergranularcorrosion of aluminium alloy.

[18] Chen S., Chen K., Peng G., Jia L., Dong P. (2012). Effect of heat treatment on strength, exfoliation corrosion and electrochemical behavior of 7085 aluminum alloy. Mater. Design.

[19] Liu S.D., Chen B., Li C.B., Dai Y., Deng Y.L., Zhang X.M. (2015). Mechanism of low exfoliation corrosion resistance due to slow quenching in high strength aluminium alloy. Corros. Sci..

[20] Sheng-dan L., Xiao-lian C., Duan-zheng Z., Yun-lai D., Xin-ming Z. (2015). Effect of solution heat treatment temperature on microstructure and properties of 6082 aluminum alloy. Chin. J. Nonferr. Metal.

[21] Edwards G.A., Stiller K., Dunlop G.L., Couper M.J. (1998). The Precipitation Sequence in Al-Mg-Si Alloys. Acta Mater..

[22] Ding L., Jia Z., Nie J.-F., Weng Y., Cao L., Chen H., Wu X., Liu Q. (2018). The structural and compositional evolution of precipitates in Al-Mg-Si-Cu alloy. Acta Mater..

[23] Guo M.X., Du J.Q., Zheng C.H., Zhang J.S., Zhuang L.Z. (2019). Influence of Zn contents on precipitation and corrosion of Al-Mg-Si-Cu-Zn alloys for automotive applications. J. Alloys Compd..

[24] Jiao W., Bing-hui L., Ya-ya Z., Zhen-hai B., Bin L. (2017). Effects of Mg-Si ratio on microstructures and properties of Al-Mg-Si alloy. Chin. J. Nonferr. Metal.

[25] Zhu S., Li Z., Yan L., Li X., Huang S., Yan H., Xiong B. (2018). Effects of Zn addition on the age hardening behavior and precipitation evolution of an Al-Mg-Si-Cu alloy. Mater. Charact..

[26] Esmaeili S., Lloyd D.J., Poole W.J. (2003). Modeling of precipitation hardening for the naturally aged Al-Mg-Si-Cu alloy AA6111. Acta Mater..

[27] Yao D., Bai Z., Qiu F., Li Y., Jiang Q. (2012). Effects of La on the age hardening behavior and precipitation kinetics in the cast Al–Cu alloy. J. Alloy Compd..

[28] Esmaeili S., Lloyd D.J., Poole W.J. (2003). A yield strength model for the Al-Mg-Si-Cu alloy AA6111. Acta Mater..

[29] Engler O., Marioara C.D., Aruga Y., Kozuka M., Myhr O.R. (2019). Effect of natural ageing or pre-ageing on the evolution of precipitate structure and strength during age hardening of Al–Mg–Si alloy AA 6016. Mater. Sci. Eng. A.

[30] Cvijović Z., Rakin M., Vratnica M., Cvijović I. (2008). Microstructural dependence of fracture toughness in high-strength 7000 forging alloys. Eng. Fract. Mech..

[31] Eckermann F., Suter T., Uggowitzer P.J., Afseth A., Schmutz P. (2008). The influence of MgSi particle reactivity and dissolution processes on corrosion in Al–Mg–Si alloys. Electrochim. Acta.

